# Supplementary material for: Resolving artefacts in voltage-clamp experiments with computational modelling: an application to fast sodium current recordings
Source: bioRxiv. 2024 Jul 24:2024.07.23.604780. Preprint. [Version 1] doi: 10.1101/2024.07.23.604780 (PMC11291073; doi:10.1101/2024.07.23.604780)
Supplement: Supplement 1 [file media-1.pdf]

Supplementary Material

Resolving artefacts in voltage-clamp experiments with a mathematical model: an application to fast sodium current recordings

Chon Lok Lei, Alexander P. Clark, Michael Clerx, Siyu Wei, Meye Bloothooft, Teun P. de Boer, David J. Christini, Trine Krogh-Madsen, and Gary R. Mirams

Author affiliations as they appear in the main article

S1 Model derivation

The derivation of the equations governing a voltage-clamp experiment *without* any compensation follows exactly as Lei et al. (2020), which is not repeated here. These include the equations for the effects of membrane capacitance, series resistance, leak current (seal resistance), pipette capacitance, and amplifier delays. Below shows the derivation of the mathematical model of how modern patch amplifiers typically compensate for them (Axon Instruments Inc., 1999; HEKA Elektronik GmbH, 2018), following the derivation from (Lei, 2020). Firstly, the voltage offset is usually estimated and compensated *prior* to adding the cell to the system, either with an automated correction estimated using *software control* or by applying manually a voltage offset such that it gives zero current when clamped at zero voltage, so the compensation circuit is not shown in our patch clamp equivalent circuits (Neher, 1995; Sigworth et al., 1995). The major source of voltage offset may be the liquid junction potential, a potential difference of $\sim 2-12\,\mathrm{mV}$ which develops when the pipette-filling solution is different from the bath solution (Neher, 1992). The adjustment is usually done by adding the theoretically estimated liquid junction potential offset to V_{off}^{*}. We can write the error in the estimate of the overall voltage offset $V_{\text{off}}^{\dagger}$ as

$$V_{\text{off}}^{\dagger} = V_{\text{off}} - V_{\text{off}}^{*}. \tag{S1.1}$$

We then simply need to replace all instances of V_{off} in the equations above with $V_{\text{off}}^{\dagger}$ to describe the effect of imperfect voltage offset compensation, and $V_{\text{off}}^{\dagger}$ is assumed to be $\mathcal{O}(10)\,\mathrm{mV}$.

Secondly, to compensate the effect of the parasitic capacitance at the electrode, an additional current is injected at the electrode to compensate for the current drawn by the parasitic capacitance. By analysing the fast capacitance compensation part, we obtain the compensation current as $C_{\text{p}}^{*}\dfrac{\mathrm{d}V_{\text{clamp}}}{\mathrm{d}t}$ where C_{p}^{*} is the amplifier's estimate of the parasitic capacitance C_{p}. Then we have

$$I_{\text{m}} = I_{\text{in}} + C_{\text{p}}^{*}\frac{\mathrm{d}V_{\text{clamp}}}{\mathrm{d}t} - I_{\text{p}} - I_{\text{leak}} - I_{\text{ion}}, \tag{S1.2}$$

and

$$I_{\text{in}} = I_{\text{ion}} + I_{\text{leak}} + C_{\text{m}}\frac{\mathrm{d}V_{\text{m}}}{\mathrm{d}t} + \left(C_{\text{p}}\frac{\mathrm{d}V_{\text{p}}}{\mathrm{d}t} - C_{\text{p}}^{*}\frac{\mathrm{d}V_{\text{clamp}}}{\mathrm{d}t}\right). \tag{S1.3}$$

This is usually known as 'C-Fast' compensation.

Thirdly, we need to consider compensation for the cell membrane capacitance C_{m}. Usually the effect of C_{m} is reduced by a hardware 'C-Slow' compensation, using a similar circuit to the 'C-Fast' compensation discussed above (Sigworth et al., 1995; Sigworth, 1995a). However, since the value of C_{m} can reach 100 pF in some cell types, and capacitor sizes can be limited, the 'C-Slow' compensation is sometimes performed as a post-processing step by the amplifier control software rather than using built-in amplifier hardware (Weerakoon et al., 2010). In either case, the full capacitance compensation can be written as

$$I_{\text{in}} = I_{\text{ion}} + I_{\text{leak}} + \left(C_{\text{m}}\frac{\mathrm{d}V_{\text{m}}}{\mathrm{d}t} - C_{\text{m}}^{*}\frac{\mathrm{d}V_{\text{est}}}{\mathrm{d}t}\right) + \left(C_{\text{p}}\frac{\mathrm{d}V_{\text{p}}}{\mathrm{d}t} - C_{\text{p}}^{*}\frac{\mathrm{d}V_{\text{clamp}}}{\mathrm{d}t}\right), \tag{S1.4}$$

where C_m^* is the amplifier (or user's) estimate of the membrane capacitance C_m, and V_est is given below.

Finally, in voltage clamp, we want V_m to approach V_cmd as quickly as possible. However, there are two effects introduced by R_s, the first one causes V_m to deviate from V_cmd and the second slows down V_m's approach to V_cmd. The first effect is caused by $(I_\mathrm{ion} + I_\mathrm{leak})$, which can be reduced through a series resistance compensation (Sigworth et al., 1995; Sigworth, 1995a; Weerakoon et al., 2009). By analysing the series resistance compensation part, instead of clamping to V_cmd, it is set to $V_\mathrm{cmd} + \alpha R_\mathrm{s}^* I_\mathrm{out}$, where R_s^* is the machine estimation of the series resistance R_s, and α is the requested proportion of series resistance compensation (a machine setting, typically 70–85 %).

The second effect is caused by the product of the series resistance R_s and the membrane capacitance C_m, i.e. the membrane assess time constant τ_a, which can be reduced through a compensation termed "supercharging" (Sigworth, 1995b). We set the clamping voltage to have a large overshoot (hence the name "supercharging") proportional to $\alpha R_\mathrm{s}^* C_\mathrm{m}^* \,\mathrm{d}V_\mathrm{est}/\mathrm{d}t$, where

$$\frac{\mathrm{d}V_\mathrm{est}}{\mathrm{d}t} = \frac{1}{R_\mathrm{s}^* C_\mathrm{m}^*}\left(V_\mathrm{cmd} + \alpha R_\mathrm{s}^* C_\mathrm{m}^* \frac{\mathrm{d}V_\mathrm{est}}{\mathrm{d}t} - V_\mathrm{est}\right) \tag{S1.5}$$

$$= \frac{V_\mathrm{cmd} - V_\mathrm{est}}{(1-\alpha) R_\mathrm{s}^* C_\mathrm{m}^*}, \tag{S1.6}$$

according to Sigworth (1995b, Figure 18). The effect of the overshooting is to charge the membrane capacitance quickly. Including all the compensations, V_clamp becomes

$$\frac{\mathrm{d}V_\mathrm{clamp}}{\mathrm{d}t} = \frac{1}{\tau_\mathrm{sum}}\left(\left(V_\mathrm{cmd} + \alpha R_\mathrm{s}^*\left(I_\mathrm{out} + C_\mathrm{m}^* \frac{\mathrm{d}V_\mathrm{est}}{\mathrm{d}t}\right)\right) - V_\mathrm{clamp}\right), \tag{S1.7}$$

to counterbalance the two effects caused by the series resistance. Note that the supercharging correction is particularly important when measuring big, very fast currents such as I_Na which has a time-to-peak within $\sim$5 ms, however this correction poses almost no issue when analysing smaller, slower currents, for example I_Kr.

Supplementary Figures

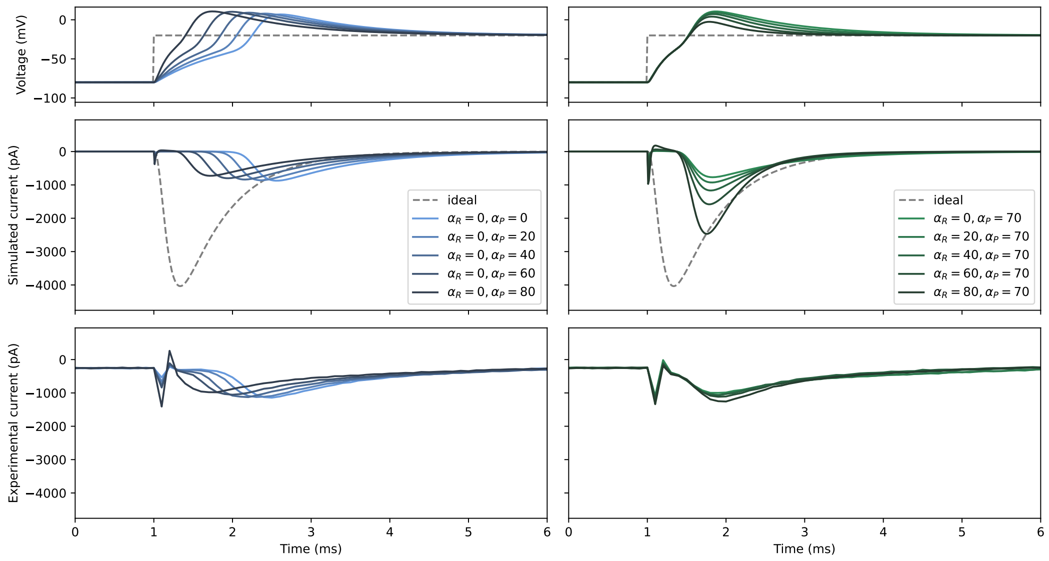

Figure S1: Forward sensitivity analysis of the experimental artefact of fast sodium current using Gray and Franz (2020) and hiPSC-CMs (cell 0).

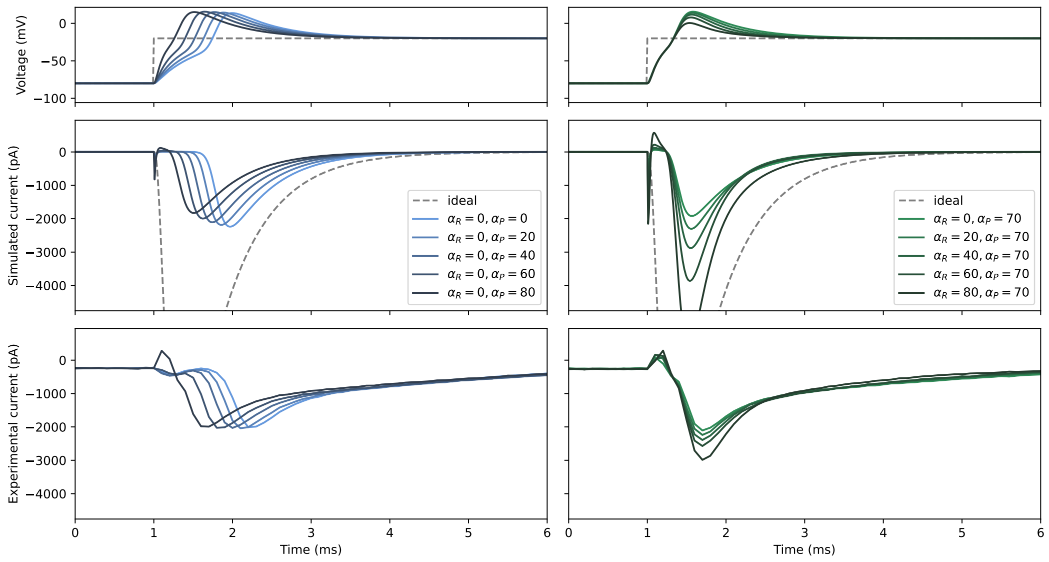

Figure S2: Forward sensitivity analysis of the experimental artefact of fast sodium current using Gray and Franz (2020) and hiPSC-CMs (cell 1; same cell as in the main text figure).

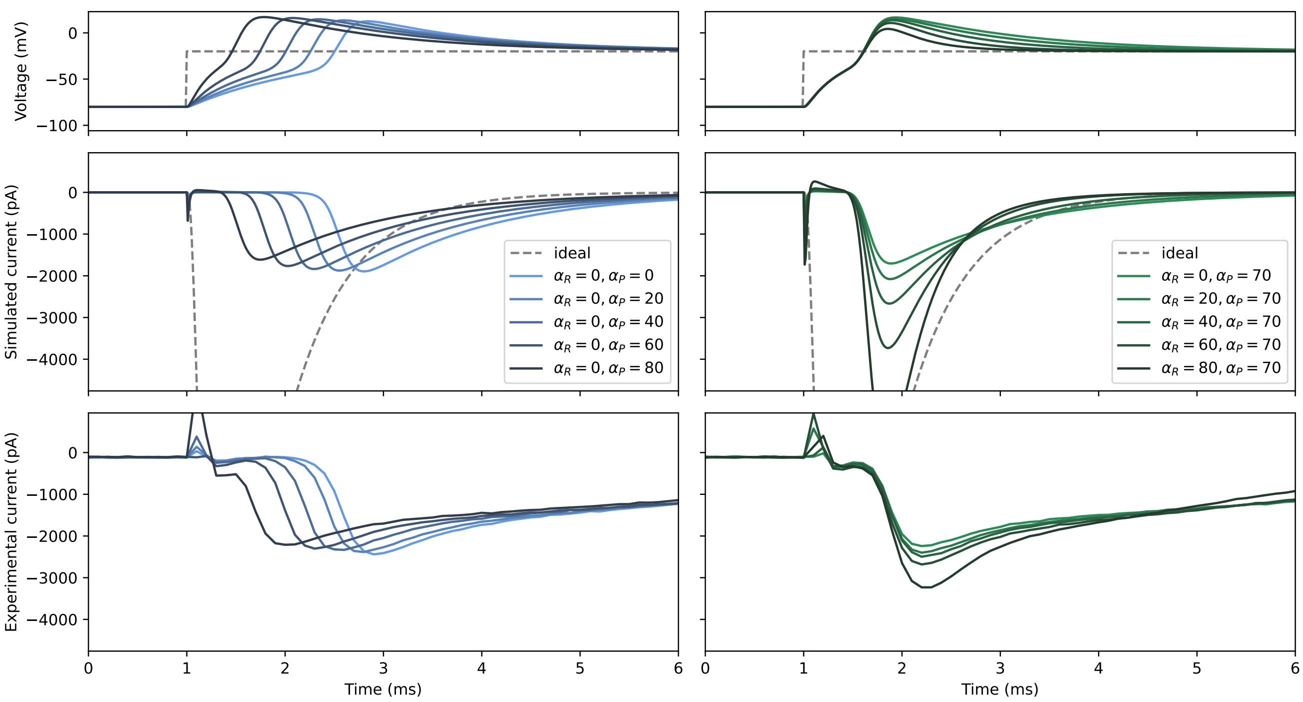

Figure S3: Forward sensitivity analysis of the experimental artefact of fast sodium current using Gray and Franz (2020) and hiPSC-CMs (cell 2).

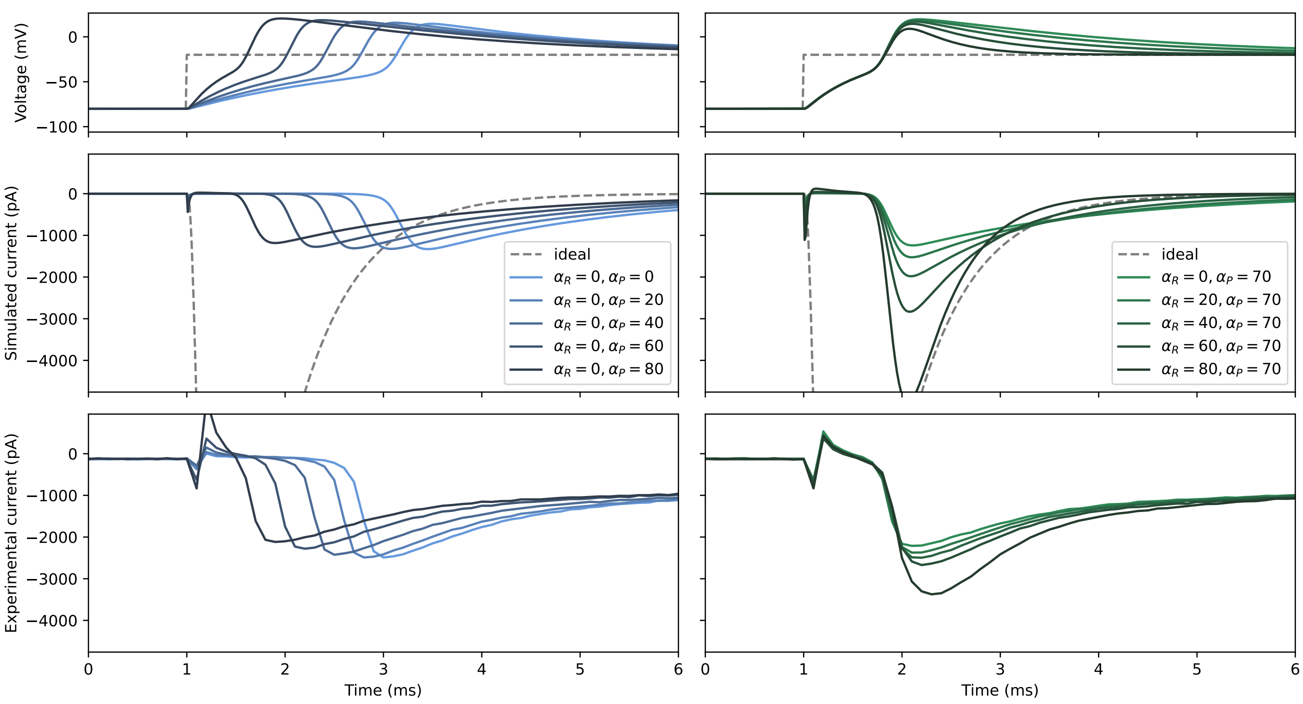

Figure S4: Forward sensitivity analysis of the experimental artefact of fast sodium current using Gray and Franz (2020) and hiPSC-CMs (cell 3).

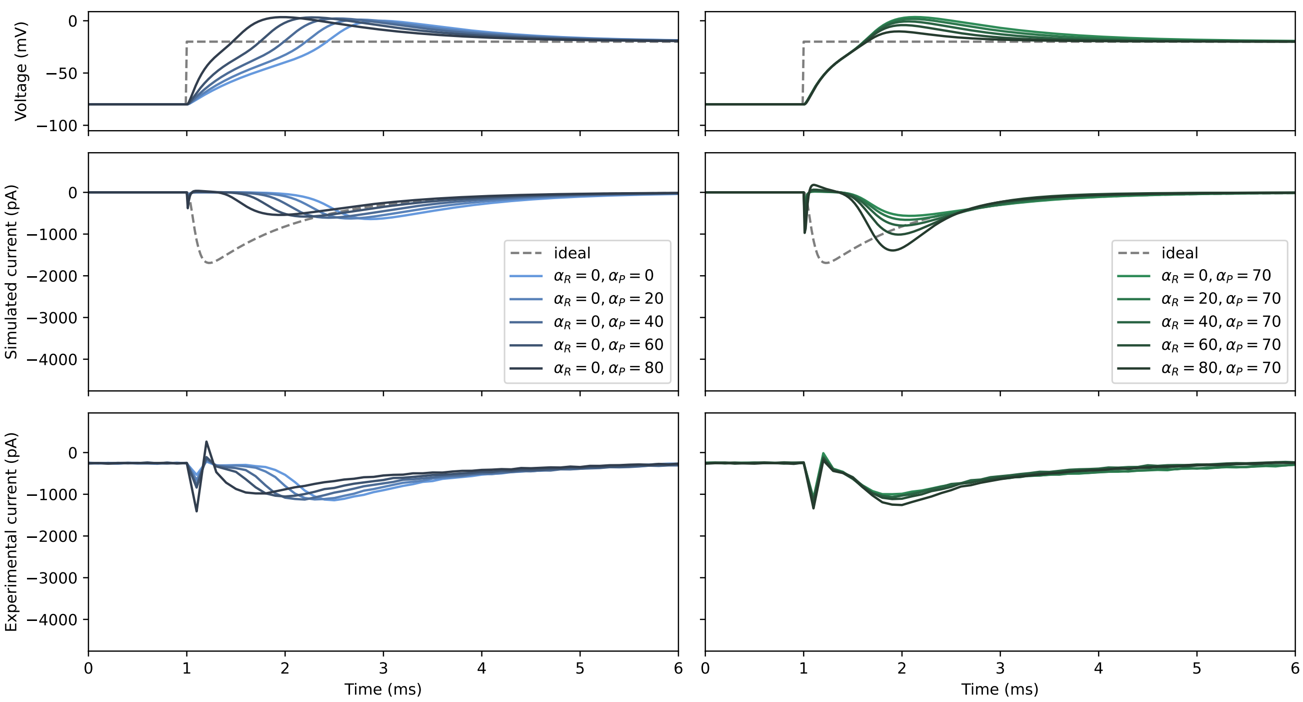

Figure S5: Forward sensitivity analysis of the experimental artefact of fast sodium current using Paci et al. (2020) and hiPSC-CMs (cell 0).

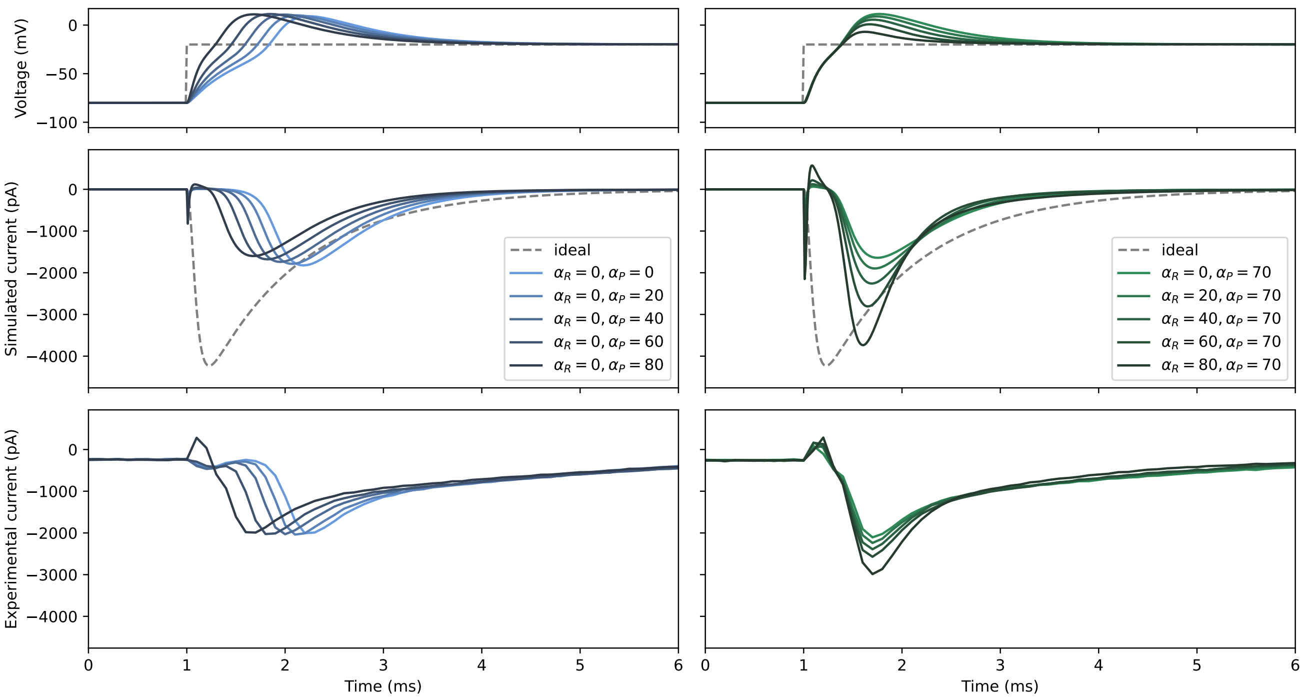

Figure S6: Forward sensitivity analysis of the experimental artefact of fast sodium current using Paci et al. (2020) and hiPSC-CMs (cell 1; same cell as in the main text figure).

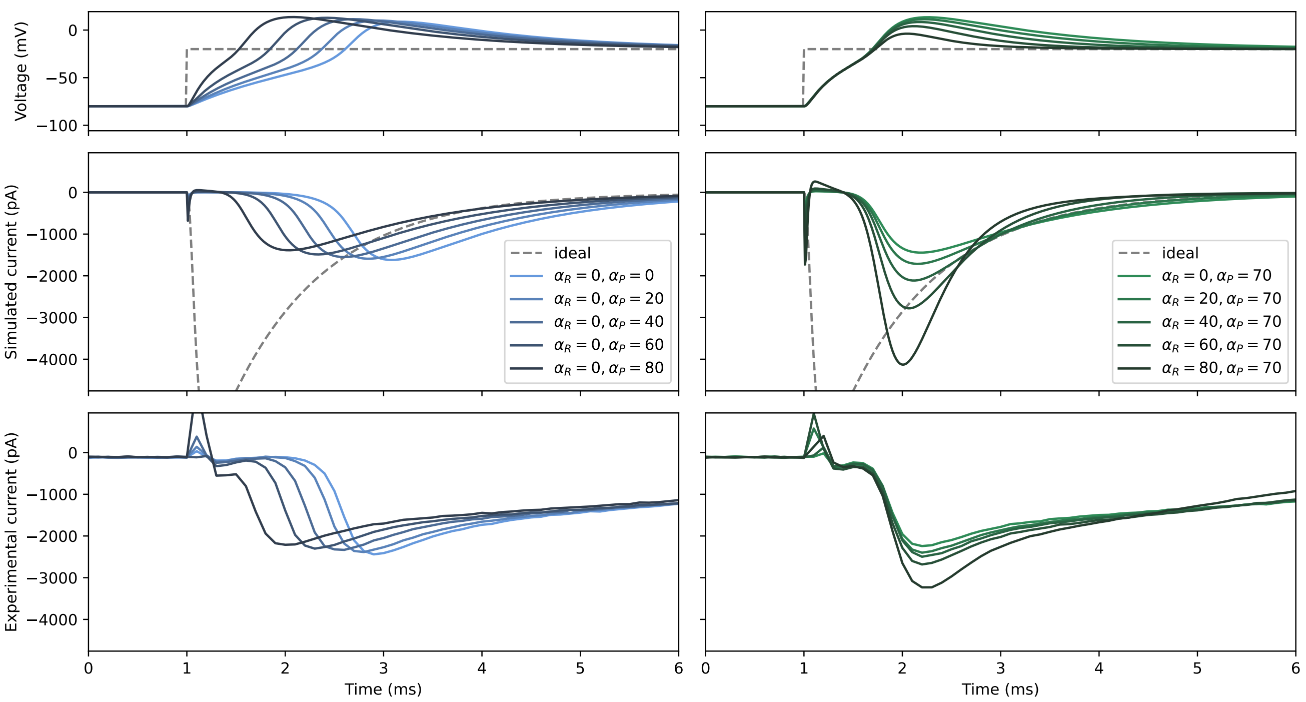

Figure S7: Forward sensitivity analysis of the experimental artefact of fast sodium current using Paci et al. (2020) and hiPSC-CMs (cell 2).

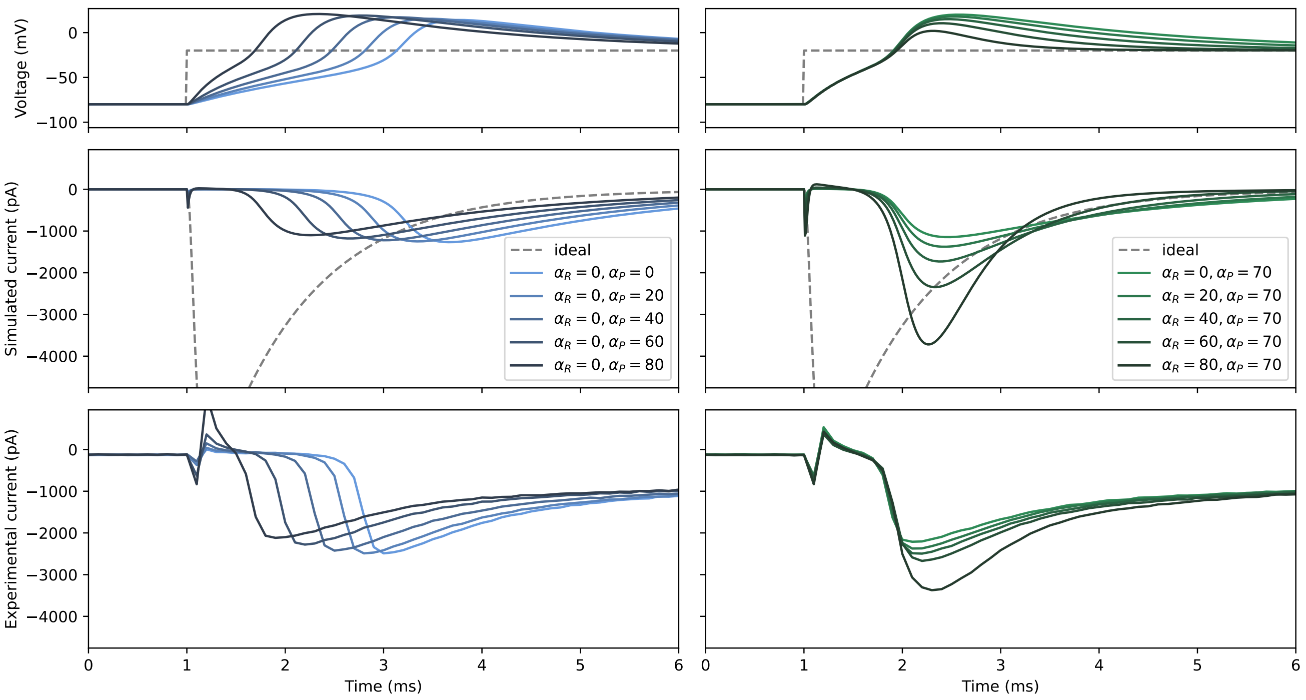

Figure S8: Forward sensitivity analysis of the experimental artefact of fast sodium current using Paci et al. (2020) and hiPSC-CMs (cell 3).

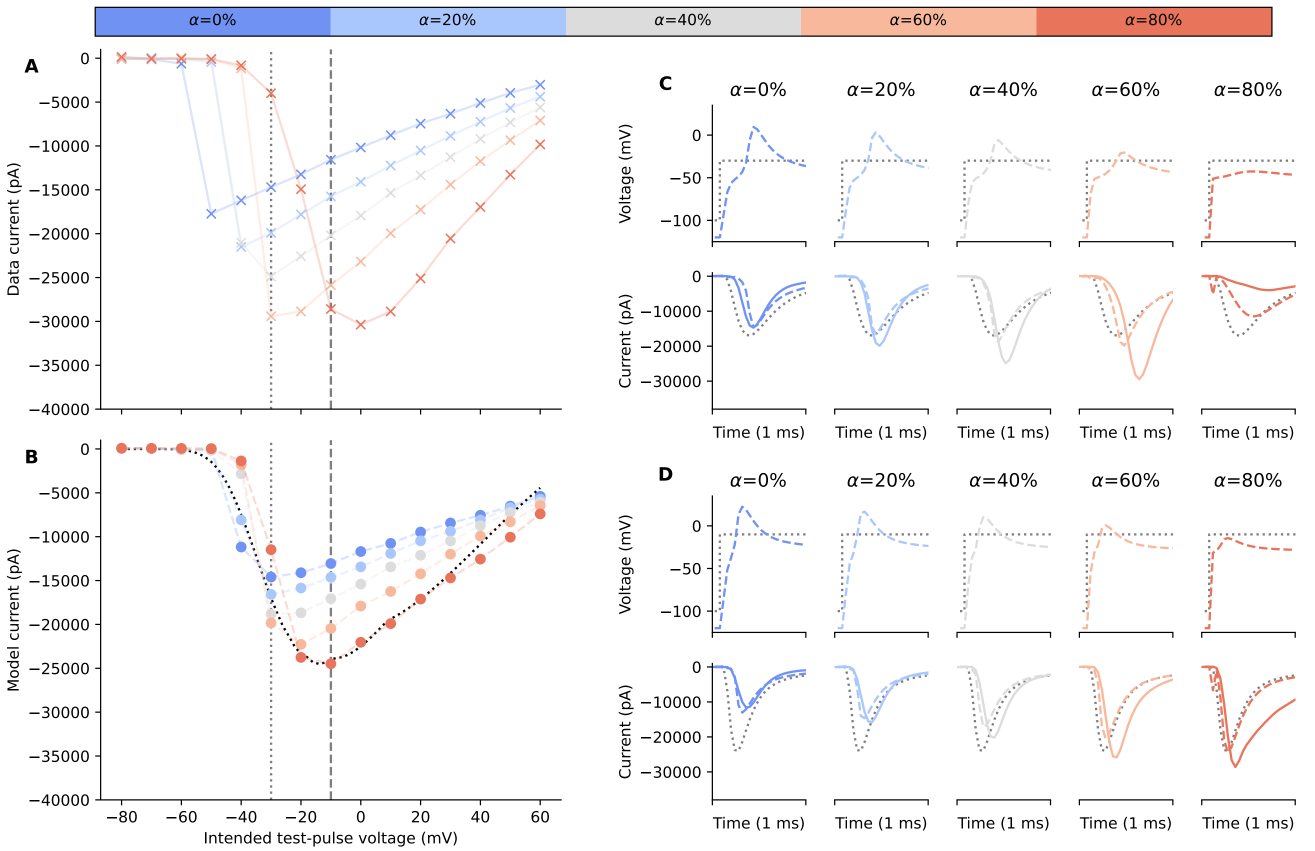

Figure S9: Correction of fast sodium current experiments (cell 1) using the experimental artefact model.

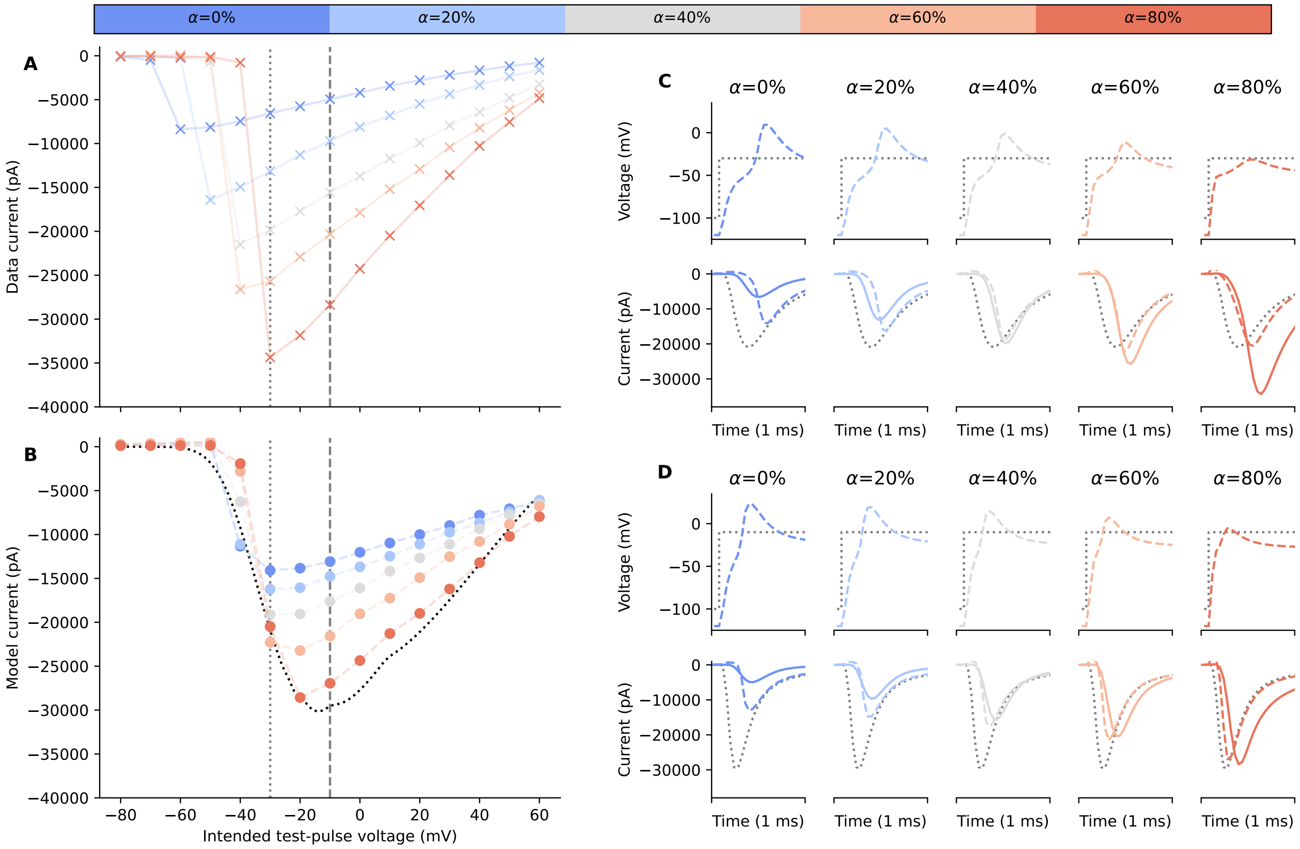

Figure S10: Correction of fast sodium current experiments (cell 2) using the experimental artefact model.

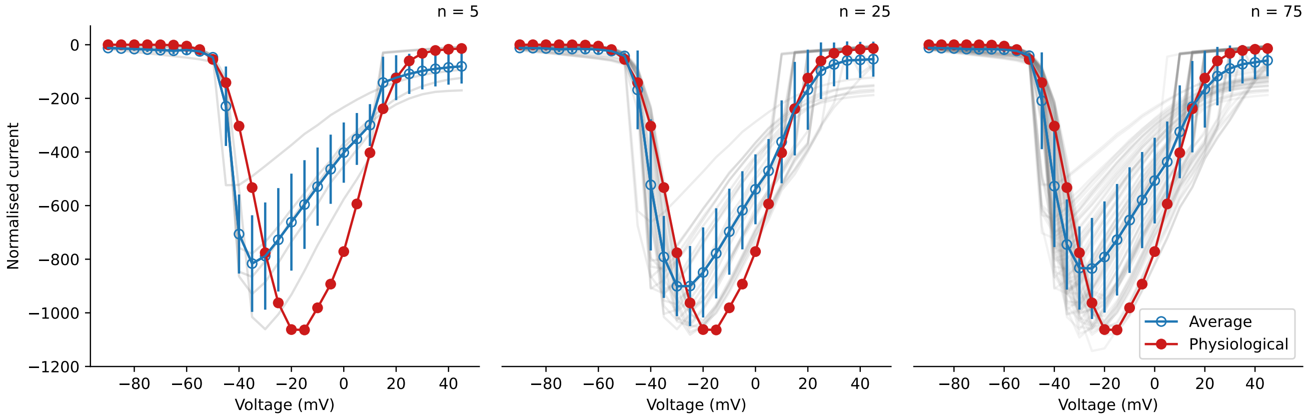

Figure S11: Consequences of averaging fast sodium current normalised I-V curves of multiple runs of experiments, with the average I-V curve shown in blue (averaged from transparent grey lines) and the artefact-free physiological I-V curve shown in red. Left to right shows different number of repeats, $n = 5$, 25, and 75, respectively. Error bars show the standard deviation of the data.

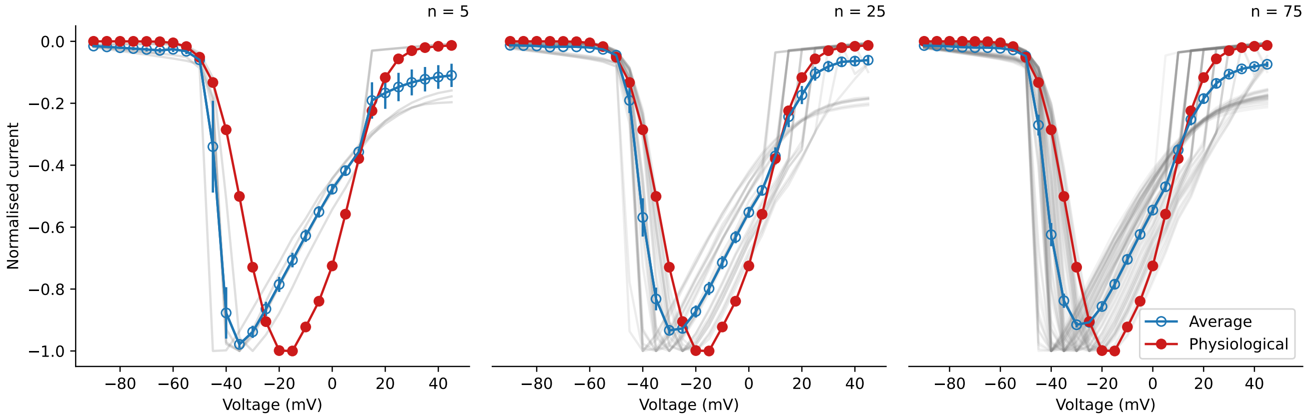

Figure S12: Consequences of averaging fast sodium current normalised I-V curves of multiple runs of experiments, with the average I-V curve shown in blue (averaged from transparent grey lines) and the artefact-free physiological I-V curve shown in red. Left to right shows different number of repeats, $n = 5$, 25, and 75, respectively. Error bars show the standard error of mean (SEM).

References

Axon Instruments Inc. (1999). Axopatch 200B patch clamp theory and operation. https://www.autom8.com/wp-content/uploads/2016/07/Axopatch-200B.pdf.

Gray, R. A. and Franz, M. R. (2020). A model for human action potential dynamics in vivo. *American Journal of Physiology-Heart and Circulatory Physiology*, 318(3):H534–H546.

HEKA Elektronik GmbH (2018). EPC 10 USB hardware manual version 2.8. http://www.heka.com/downloads/hardware/manual/m_epc10.pdf.

Lei, C. L. (2020). *Model-Driven Design and Uncertainty Quantification for Cardiac Electrophysiology Experiments*. PhD thesis, University of Oxford.

Lei, C. L., Clerx, M., Whittaker, D. G., Gavaghan, D. J., De Boer, T. P., and Mirams, G. R. (2020). Accounting for variability in ion current recordings using a mathematical model of artefacts in voltage-clamp experiments. *Philosophical Transactions of the Royal Society A*, 378(2173):20190348.

Neher, E. (1992). Correction for liquid junction potentials in patch clamp experiments. In *Methods in Enzymology*, volume 207 of *Ion Channels*, chapter 6, pages 123–131. Academic Press, Cambridge, MA.

Neher, E. (1995). Voltage offsets in patch-clamp experiments. In Sakmann, B. and Neher, E., editors, *Single-Channel Recording*, chapter 6, pages 147–153. Springer, Boston, MA, 2nd edition.

Paci, M., Passini, E., Klimas, A., Severi, S., Hyttinen, J., Rodriguez, B., and Entcheva, E. (2020). All-optical electrophysiology refines populations of in silico human ipsc-cms for drug evaluation. *Biophysical Journal*, 118(10):2596–2611.

Sigworth, F. (1995a). Design of the EPC-9, a computer-controlled patch-clamp amplifier. 1. Hardware. *Journal of Neuroscience Methods*, 56(2):195–202.

Sigworth, F., Affolter, H., and Neher, E. (1995). Design of the EPC-9, a computer-controlled patch-clamp amplifier. 2. Software. *Journal of Neuroscience Methods*, 56(2):203–215.

Sigworth, F. J. (1995b). Electronic design of the patch clamp. In Sakmann, B. and Neher, E., editors, *Single-Channel Recording*, chapter 6, pages 95–127. Springer, Boston, MA, 2nd edition.

Weerakoon, P., Culurciello, E., Klemic, K. G., and Sigworth, F. J. (2009). An integrated patch-clamp potentiostat with electrode compensation. *IEEE Transactions on Biomedical Circuits and Systems*, 3(2):117–125.

Weerakoon, P., Culurciello, E., Yang, Y., Santos-Sacchi, J., Kindlmann, P. J., and Sigworth, F. J. (2010). Patch-clamp amplifiers on a chip. *Journal of Neuroscience Methods*, 192(2):187–192.
